# Cinnamon and Hop Extracts as Potential Immunomodulators for Severe COVID-19 Cases

**DOI:** 10.3389/fpls.2021.589783

**Published:** 2021-02-26

**Authors:** Kurt Lucas, Janine Fröhlich-Nowoisky, Nicole Oppitz, Maximilian Ackermann

**Affiliations:** ^1^Multiphase Chemistry Department, Max Planck Institute for Chemistry, Mainz, Germany; ^2^Institute of Pathology and Molecular Pathology, Helios University Clinic Wuppertal, University of Witten/Herdecke, Wuppertal, Germany; ^3^Institute of Functional and Clinical Anatomy, University Medical Center of the Johannes Gutenberg University Mainz, Mainz, Germany

**Keywords:** COVID-19, Ceylon cinnamon, hops, anti-inflammatory (activity), angiogenesis inhibition

## Introduction

Despite intense focus, so far no effective treatment has been developed for severe cases of COVID-19. SARS-CoV-2 infection results in a multisystem hyperinflammatory syndrome with acute respiratory distress syndrome (ARDS), acute kidney failure, and cardiovascular and neurological complications (Wang et al., 2020). Severe cases of this condition are characterized by a “cytokine storm” and rampant inflammation (Renu et al., 2020; Ye et al., 2020). The hyperinflammation is associated with the generation and release of reactive oxygen and nitrogen species (ROS/RNS), which can further amplify inflammation (Lucas and Maes, 2013). Histopathological observation of COVID-19 has revealed diffuse alveolar damage with vascular endothelialitis, thrombosis, and intussusceptive angiogenesis (Ackermann et al., 2020). The angiocentric inflammation is not limited to the COVID-19-induced lung injury but also involves prolonged inflammation in other organs, such as the liver, brain, heart, or the gut (Ackermann et al., 2020; Wang et al., 2020). Any treatment that could limit the “cytokine storm,” reduce ROS/RNS production, and counteract the formation of thrombosis would be highly attractive, and, in the best-case scenario, such a treatment would additionally interfere with viral replication. Recently, a preliminary study reported that the administration of dexamethasone, a corticosteroid with anti-inflammatory effects, could elicit a 30% reduction in mortality for patients receiving invasive mechanical ventilation (Horby et al., 2020).

In a previous study, we screened a panel of 99 ethanolic herbal extracts for their anti-inflammatory properties. Hop (*Humulus lupulus*, cones) and Ceylon cinnamon (*Cinnamomum verum* alias *C. zeylanicum*, bark) extracts were found to elicit particularly drastic reductions in activation of the transcription factor NF-κB (nuclear factor kappa-light-chain-enhancer of activated B cells), a key regulator of pro-inflammatory cytokines (Schink et al., 2018b).

The literature search was mainly performed in MedLine. In a first round, we looked for studies describing anti-inflammatory effects for Ceylon cinnamon, hops, and their major compounds. In a second approach, we then searched for clinical studies testing the efficacy of the plants in treating human disease.

## HOPS

Hops, the seed cones of the plant *Humulus lupulus* from the family Cannabaceae, contain several pharmaceutically active compounds, such as humulone, lupulone, and xanthohumol (Gerhäuser, 2005; Knez Hrncic et al., 2019; Lin et al., 2019). Crude hop extracts as well as individual compounds have been described to exert anti-viral effects against several DNA and RNA viruses (Buckwold et al., 2004; Fuchimoto et al., 2013). For example, humulone, the most important bitter acid of hops, can suppress replication of the respiratory syncytial virus (RSV alias Human orthopneumovirus) in cell culture by disturbing the formation of viral filaments (Fuchimoto et al., 2013). Xanthohumol from hops showed synergistic effects with IFN-α in the treatment of bovine viral diarrhea virus (BVDV), and a combination of the two substances was more effective than IFN-α or xanthohumol alone (Zhang et al., 2010).

Scientific reports on the biological effects of hops and hop compounds are not limited to anti-viral properties; anti-bacterial activity, anti-fungal properties, and anti-malarial action have also been reported (Gerhäuser, 2005; Cermak et al., 2017; Weber et al., 2019). Lupulone and xanthohumol exhibit synergistic effects with selected clinically used antibiotics (Natarajan et al., 2008).

Hops also counteract inflammation; humulone can exert an anti-inflammatory effect on the TNF-induced expression of cyclooxygenase with effective doses that are in the same order of magnitude as dexamethasone (Yamamoto et al., 2000). Moreover, humulone can inhibit Toll-like receptor 4 (TLR4) and NF-κB signaling (Yamamoto et al., 2000; Fu et al., 2016; Schink et al., 2018b). In an animal model, topically applied humulone was shown to inhibit NF-κB, AP-1, and mitogen-activated protein kinases (MAPKs) (Lee et al., 2007). Xanthohumol from hops was found to reduce the expression of IL-1β, IL-6, IL-8, and TNF in virus-infected and LPS-stimulated porcine primary alveolar macrophages (Liu et al., 2019), and hop-derived humulone and lupulone were shown to mitigate the expression of IL-6 (Weber et al., 2019).

Hop extracts are further effective in counteracting oxidative and nitrosative stress; hop compounds were shown to mitigate neural nitric oxide synthase (nNOS) activity and 3-morpholinosydnonimine (SIN-1)-induced oxidation of LDL (Stevens et al., 2002). The transcription factor nuclear factor erythroid 2-related factor 2 (NRF2) is a key regulator of the expression of antioxidant genes (Yamamoto et al., 2018). Hop compounds and especially xanthohumol were shown to activate NRF2 in different studies (Dietz et al., 2005; Lee et al., 2011; Yao et al., 2015). A small, placebo-controlled, clinical study showed that a low-intake dose of 12 mg/day xanthohumol decreased oxidative stress-induced DNA damage (Ferk et al., 2016).

Xanthohumol was also shown to be effective in preventing thrombosis in animal models (Xin et al., 2017). In contrast to what has been observed for blood thinners, no increased bleeding was seen when the substance was administrated orally in two doses of 10 mg/kg xanthohumol per day for 60 days (Xin et al., 2017). The antioxidant effects of xanthohumol were also confirmed in this same study (Xin et al., 2017). Further, hops exert anti-fibrogenic effects *in vitro*, and specifically xanthohumol was shown to possess this property *in vivo* (Dorn et al., 2012; Saugspier et al., 2012).

## Ceylon Cinnamon

The genus *Cinnamomum* belongs to the family Lauraceae and comprises more than 100 species in the NCBI Taxonomy Database. Here, we review specifically Ceylon cinnamon (*Cinnamomum verum* alias *C. zeylanicum*, NCBI:txid128608), which we have used in our studies (Schink et al., 2018a,b; Ose et al., 2020). Generally, Chinese cassia or Chinese cinnamon (*Cinnamomum cassia* alias *Cinnamomum aromaticum*) is commercially sold as a spice for foods and does not represent a risk to human health *per se* (Oketch-Rabah et al., 2018). However, it can comprise high amounts of coumarin, which is highly hepatotoxic in higher doses. The German Federal Institute for Risk Assessment (BfR) therefore recommends that Cassia cinnamon with high coumarin content be consumed only at moderate levels. The differences between Ceylon cinnamon and Cassia have been reviewed recently (Oketch-Rabah et al., 2018).

Ethanolic extracts of Ceylon cinnamon possess anti-inflammatory activity and antagonize TLR2 and TLR4 activation in a dose-dependent manner with minimal effects on viability in cell culture (Kanuri et al., 2009; Schink et al., 2018b). We identified several active compounds in these extracts, amongst others, trans-cinnamaldehyde, cinnamic acid, cinnamyl alcohol, cinnamyl methyl ether, p-cymene, methyl salicylate, 1-tetradecanol (myristyl alcohol), and benzoic acid. We found synergy among the anti-inflammatory properties of the different compounds: the efficacy of the complex mixture was greater than those of the pure active compounds of cinnamon (Schink et al., 2018a).

Cinnamaldehyde is an effective NRF2 inducer (Long et al., 2015) and acts in this way to detoxify ROS/RNS (Wondrak et al., 2010). Further, cinnamaldehyde can inhibit angiogenesis and metastasis via mitigation of the PI3K/Akt pathway (Patra et al., 2019). A specific inhibition of VEGFR2 kinase and of angiogenesis was shown for a water-based extract from Ceylon cinnamon (Lu et al., 2010).

In an animal model, an extract of Ceylon cinnamon was shown to protect the aorta from dexamethasone-induced atherosclerosis and minimized the atherogenic risk (Nayak et al., 2017).

## Retrieved Results from Clinical Studies

We searched for clinical studies on Cinnamon using the search term “Ceylon Cinnamon OR Cinnamaldehyde,” while for clinical studies on hops we used the search term “Humulone OR Lupulone OR Xanthohumol OR hops” (Tables 2, 3). For both search terms, we restricted the results to “Clinical Trial.” For Cinnamon we obtained 123 hits, from which we excluded 57 hits, as cinnamon was not used to treat human disease in these studies. The ethanolic extract, as used in cell culture experiments, was not directly given to patients in any of the clinical studies. Rather, for the majority of clinical studies with Ceylon cinnamon, powder was administered in an encapsulated form; the clear advantage of administering Ceylon cinnamon in this way is that the formulation does not comprise ethanol. Since we cannot give here an appropriate dosage of cinnamon for the treatment of COVID-19, we refer to clinical studies that have used cinnamon to treat other conditions. Thirty of the clinical Cinnamon studies deal with diabetes, glucose levels, and insulin tolerance, five with polycystic ovary syndrome, and three with overweight and obesity. We could not find any clinical studies on “cytokine storms,” but we hypothesize that the strong anti-inflammatory properties of Ceylon cinnamon may mitigate this complication. Ranasinghe et al. evaluated the safety of Ceylon cinnamon in healthy adults, concluding that there were no significant side effects and toxicity of Ceylon cinnamon for the dosages applied (Ranasinghe et al., 2017b).

When searching for hops and compounds found in hops, we obtained 92 hits for clinical studies, from which we excluded 50 as non-matching. This is because many hits refer to the verb “to hop,” i.e., “to jump.” Eight studies are linked to sleep disorders, six to adiposity and metabolic syndrome, six to menopause, and two clinical studies are on anti-bacterial effects. Often hops are given orally as an extract (water, oil), which is frequently administered in combination with other drugs such as valerian. One study concerns specifically the use of *iso*-alpha-acids from hops, which include humulone, to dampen inflammation in knee osteoarthritis (Hall et al., 2008).

Taken together, among the so-far performed clinical studies on Ceylon cinnamon and hops, we could not find any that specifically described efficacy in preventing the “cytokine storm” or sepsis. However, results from diverse cell culture experiments make it likely that hops and Ceylon cinnamon may exert these effects.

## Discussion

Both hop and cinnamon extracts have been shown to exert several anti-inflammatory functions (Yamamoto et al., 2000; Schink et al., 2018a,b). For instance, both can dampen the release of pro-inflammatory cytokines (Lee et al., 2007; Schink et al., 2018b; Liu et al., 2019; Weber et al., 2019). Moreover, hop and cinnamon extracts can inhibit angiogenesis, thrombosis, and vascular endothelialitis (Dorn et al., 2012; Saugspier et al., 2012; Xin et al., 2017; Patra et al., 2019). Further, these herbal extracts can activate the key regulator of the antioxidant response, NRF2, which mitigates the ROS/RNS production generally associated with inflammation (Dietz et al., 2005, 2013; Wondrak et al., 2010; Lee et al., 2011; Pinto et al., 2014; Long et al., 2015; Yao et al., 2015). Taken together, we suggest that hop and Ceylon cinnamon extracts may ameliorate complications that are associated with severe cases of COVID-19 and that testing both extracts, either alone or in combination, and particularly as a supplemental treatment to other medications, might be a promising therapeutic approach. If the preliminary results for dexamethasone can be confirmed, this glucocorticoid may be widely used to treat cases of COVID-19 (Horby et al., 2020). Supplementation with Ceylon cinnamon extract could then ameliorate the potential side effects of dexamethasone such as atherosclerosis (Nayak et al., 2017). Hop extracts exert anti-viral effects against some virus strains, but whether these extracts are also effective against SARS-CoV-2 has not yet been experimentally verified (Buckwold et al., 2004; Zhang et al., 2010; Fuchimoto et al., 2013).

Pneumonia caused by bacteria is a frequent complication after artificial ventilation (Póvoa et al., 2020; Wu et al., 2020; Zhang et al., 2020). The anti-bacterial effects of hops (Gerhäuser, 2005; Natarajan et al., 2008; Cermak et al., 2017; Weber et al., 2019) and Ceylon cinnamon (Ranasinghe et al., 2013; Vasconcelos et al., 2018; Doyle and Stephens, 2019) could act preventatively in such cases. As they are derived from common foodstuffs, both hop and Ceylon cinnamon extracts can be regarded as safe. Of course, an allergy against a hop or cinnamon ingredient or alcohol intolerance would contraindicate their intake.

It has become clear that many COVID-19 patients suffer from inflammatory complications (Heneka et al., 2020; Portincasa et al., 2020). We suggest that treatment with the here-discussed extracts could also mitigate such complications. Should cinnamon and hops prove to exert positive effects in the treatment of COVID-19, they would be readily available at low cost and can be produced at multi-ton scales. In western medicine, it is common to use pure substances rather than less well-defined herbal extracts. Of course, the individual compounds as listed in Table 1 can be used for treatment, but a certain loss of synergy may result (Schink et al., 2018a).

**Table 1:** Active compounds from hops and Ceylon cinnamon.

**Plant**	**Active compound**	**References**
Hops	humulone	Palamand and Aldenhoff, 1973; Gerhäuser, 2005; Knez Hrncic et al., 2019; Lin et al., 2019
	lupulone	Palamand and Aldenhoff, 1973; Gerhäuser, 2005; Knez Hrncic et al., 2019; Lin et al., 2019
	xanthohumol	Palamand and Aldenhoff, 1973; Gerhäuser, 2005; Fu et al., 2016; Knez Hrncic et al., 2019; Lin et al., 2019
Ceylon cinnamon	trans-cinnamaldehyde	Schink et al., 2018a; Vasconcelos et al., 2018
	cinnamic acid	Schink et al., 2018a; Vasconcelos et al., 2018
	cinnamyl alcohol	Schink et al., 2018a

**Table 2:** Selected clinical studies on Ceylon cinnamon.

**Disease/condition or observed effect**	**Dosage form**	**References**
**Ceylon Cinnamon**		
type 2 diabetes	encapsulated cinnamon power	Khan et al., 2003; Mang et al., 2006; Crawford, 2009; Akilen et al., 2010; Wainstein et al., 2011; Talaei et al., 2017; Mirmiran et al., 2019; Zare et al., 2019
	cinnamon extract	Lu et al., 2012; Ranasinghe et al., 2017a
	cinnamon in black tea	Azimi et al., 2016
polycystic ovary syndrome	encapsulated cinnamon power	Kort and Lobo, 2014; Borzoei et al., 2018; Hajimonfarednejad et al., 2018
dysmenorrhea (painful periods)		Jahangirifar et al., 2018
metabolic syndrome		Gupta Jain et al., 2017
migraine attacks and inflammatory markers		Zareie et al., 2020
pharmacodynamic properties and safety		Ranasinghe et al., 2017b
postmenopausal type 2 Diabetes		Vanschoonbeek et al., 2006
overweight or obese pre-diabetic subjects		Liu et al., 2015
non-alcoholic fatty liver disease		Askari et al., 2014
Ceylon cinnamon does not affect postprandial plasma glucose or insulin		Wickenberg et al., 2012
overweight or obese subjects	cinnamon extract	Roussel et al., 2009
effect on electrocardiographic parameters		Pender et al., 2018
perineal pain and healing of episiotomy		Mohammadi et al., 2014
*Helicobacter pylori*		Nir et al., 2000
type 1 diabetes	cinnamon pill	Altschuler et al., 2007
overactive bladder	cinnamon patch	Chen et al., 2021
postprandial (after meals) capillary blood glucose level	cinnamon tea	Bernardo et al., 2015

**Table 3:** Selected clinical studies on hops.

**Disease/condition or observed effect**	**Dosage form**	**References**
**Hops**		
quality of sleep	standardized extracts of *Valeriana officinalis, Passiflora incarnate*, and *Humulus lupulus*	Maroo et al., 2013
	linolenic and linoleic acids in association with *Humulus lupulus* extract.	Cornu et al., 2010
	valerian/hop extract combination	Müller-Limmroth and Ehrenstein, 1977; Koetter et al., 2007; Dimpfel and Suter, 2008
Vigilance	tablets containing valerian and hops	Gerhard et al., 1996
menopausal symptoms	hop extract	Erkkola et al., 2010; van Breemen et al., 2020
	hop tablets	Aghamiri et al., 2016
appetite suppression	hop flower extract suspended in canola oil	Walker et al., 2019
body fat	matured hop extract	Morimoto-Kobayashi et al., 2016; Suzuki et al., 2018
dental plaque regrowth	hop bract polyphenols	Shinada et al., 2007
clinical safety and efficacy	combination of iso-alpha acids from hops, rosemary, and oleanolic acid	Minich et al., 2007
self-reported depression, anxiety, and stress levels	dry hop extract	Kyrou et al., 2017
overactive bladder	combination of seed oil from Uromedic pumpkin, Rhus aromatica (bark extract, and hop cone extract)	Gauruder-Burmester et al., 2019
intestinal conversion of isoxanthohumol in 8-prenylnaringenin	dose of isoxanthohumol	Possemiers et al., 2006
endothelial functions	isomerized hop extract	Tomita et al., 2017

In conclusion, we recommend future experiments on hops and Ceylon cinnamon to evaluate their potential in limiting overshooting immune reactions in COVID-19. We work mainly with ethanolic extracts in cell culture. However, encapsulated cinnamon powder or water extracts of hops may be better suited for administration to patients. We suggest that appropriate doses for treatment of COVID-19 patients may be determined with reference to the clinical studies that have used hops and Ceylon cinnamon to treat other conditions.

## Author Contributions

KL and MA initiated the idea of the review and were involved in the manuscript writing. J-FN was involved in manuscript refinement. NO was involved in the literature search. All authors contributed to the article and approved the submitted version.

## Conflict of Interest

The authors declare that the research was conducted in the absence of any commercial or financial relationships that could be construed as a potential conflict of interest.
